# Low incidence of malignancy in patients with suspected polymyalgia rheumatica or giant cell arteritis, examined with FDG-PET/CT

**DOI:** 10.3389/fmed.2024.1309905

**Published:** 2024-02-21

**Authors:** Tanja Fromberg Gorlen, Jane Maestri Brittain, Mikkel Østergaard, Barbara Malene Fischer, Uffe Møller Døhn, Lene Terslev

**Affiliations:** ^1^Copenhagen Center for Arthritis Research, Center for Rheumatology and Spine Diseases, Centre for Head and Orthopaedics, Copenhagen University Hospital – Rigshospitalet Glostrup, Copenhagen, Denmark; ^2^Department of Clinical Medicine, Faculty of Health and Medical Sciences, The University of Copenhagen, Copenhagen, Denmark; ^3^Department of Clinical Physiology & Nuclear Medicine, Copenhagen University Hospital – Rigshospitalet, Copenhagen, Denmark

**Keywords:** polymyalgia rheumatica (PMR), giant cell arteritis (GCA), FDG (18F-fluorodeoxyglucose)-PET/CT, malignancy, diagnostic examination

## Abstract

**Introduction:**

The need to systematically examine patients suspected of polymyalgia rheumatica (PMR) and giant cell arteritis (GCA) for malignancy is controversial. The aim of this study was to assess the frequency of malignancy in patients with suspected PMR and/or GCA who have been referred to a 2-deoxy-2-[^18^F]fluoro-D-glucose positron emission tomography with computed tomography (FDG-PET/CT) as part of the diagnostic investigation.

**Method:**

The records of all patients referred to FDG-PET/CT from Center for Rheumatology and Spine Diseases, Rigshospitalet, Glostrup with the suspicion of PMR and/or GCA during a two-year period, were retrospectively reviewed. Data was analyzed with descriptive statistics, and a standard incidence ratio was calculated based on background cancer incidences extracted from the NORDCAN database.

**Results:**

220 patients were included in the study. Findings suspicious of malignancy were found in 19 of the examinations, and in seven cases (3.2%), malignancy was confirmed. In three out of the seven cases the patients were diagnosed with PMR concomitantly with malignancy. The estimated standardized incidence ratio (SIR) for cancer compared to the background incidence of cancer in Denmark was 1.58 (95% CI 0.63–2.97), i.e., not statistically significant. There were no statistically significant differences in characteristics of the patients that were diagnosed with malignancy compared with those that were not.

**Conclusion:**

The frequency of malignancy in this cohort of patients with suspected PMR/GCA who underwent PET/CT was low. Our results, though based on a small cohort, do not suggest that all patients with suspected PMR/GCA should systematically be examined with FDG-PET/CT for excluding malignancy.

## Introduction

Polymyalgia rheumatica (PMR) and giant cell arteritis (GCA) are related inflammatory conditions, that may occur concomitantly (1). Classical symptoms of PMR are pain and stiffness of the shoulder girdle, the proximal muscles of the arms, neck, pelvic girdle and the proximal part of the thighs (2, 3), whereas GCA is a vasculitis of medium-sized and/or large arteries, that can affect aorta and its branches and/or the cranial arteries, especially the temporal arteries (2, 4). Both conditions can be accompanied by malaise and constitutional symptoms like fever, weight loss and fatigue, and are usually characterized by elevated inflammatory markers and rapid glucocorticoid response (2–4).

Both conditions can be difficult to diagnose. There is no gold standard available for the diagnosis of PMR, but ultrasound can demonstrate bursitis and synovitis in shoulders and hips (5), and 2-deoxy-2-[^18^F]fluoro-D-glucose positron emission tomography with computed tomography (FDG-PET/CT) can reveal FDG enhancement in bursae, entheses and joints in certain anatomical sites, such as, shoulders and hips, sternoclavicular joints, lumbar spine, and ischial tuberosities (6). Clinical guidelines do however, recommend that a variety of medical conditions, including malignancy, should be considered before making the final diagnosis (7, 8). GCA is a serious condition, and the need for a quick diagnosis is important due to the risk of severe complications such as permanent blindness. GCA can be diagnosed with the use of a temporal artery biopsy, FDG-PET/CT, ultrasound, or Magnetic Resonance Imaging (9, 10). However, the nonspecific symptoms, especially in patients without cranial features can propose a challenge for the clinician (11, 12).

A possible connection between PMR/GCA and malignancy is a subject of controversy, and in a diagnostic context, there are two relevant issues to be considered in relation to this. Firstly, malignancy may mimic PMR/GCA due to unspecific symptoms like malaise, weight loss, widespread pain, and elevated inflammatory markers. Secondly, it has been hypothesized that PMR/GCA may occasionally appear as a paraneoplastic phenomenon, and thus co-occur with malignancy (13). Previous studies have focused on malignancy in patients with already diagnosed PMR or GCA (based on classification criteria, diagnose coding, temporal artery biopsy etc.) (14–17), and have thus not included the issue of malignancy as a differential diagnosis to PMR/GCA. It remains to be established whether it is indicated to systematically examine all patients with PMR/GCA-like symptoms for detecting occult malignancy as routine part of the diagnostic work-up.

One way to investigate malignancy is by use of FDG-PET/CT, which is a hybrid imaging modality that combines the visualization of functional processes (glucose metabolism) with anatomy. Thus, it allows the detection of specific body-sites with a high glucose metabolism, such as sites of inflammation, infection, or cancer (18, 19). In Denmark, access to PET/CT-scans in the investigation of infectious and inflammatory diseases have increasingly been prioritized. Consequently, FDG-PET/CT is widely used among rheumatologists and is readily available for hospital rheumatologists in Denmark for supporting the diagnostic set-up for PMR/GCA. It is, however, a costly procedure with long patient preparation time, a substantial radiation dose, and in many countries with limited availability. Therefore the extent of its use should be carefully considered.

In order to evaluate the relevance of routine use of FDG-PET/CT for all patients with PMR/GCA-like symptoms, the aim of the current study was to assess the frequency of malignancy in patients with suspected PMR and/or GCA, referred to an FDG-PET/CT examination.

## Materials and methods

In this retrospective study, we included all those patients with suspected PMR and/or GCA, referred to an FDG-PET/CT as part of their diagnostic process from the Center of Rheumatology and Spine Diseases, Rigshospitalet, in the period 04.21.19–04.21.21. In this period, an estimate of 390 patients were seen in our department suspected of having PMR/GCA.

We included both patients with suspected PMR/GCA who were referred with suspicion of underlying malignancy and patients with suspected PMR/GCA referred for diagnostic reasons, as possible occult cancer could also occur in the latter. To identify these patients, we obtained a list of all patients referred for FDG-PET/CT examinations in the study period from our department to the Department of Clinical Physiology and Nuclear Medicine, Rigshospitalet, Copenhagen. The patient charts including the referral to FDG-PET/CT were screened to identify the examinations that were performed based on suspicion of PMR, GCA or both, with and without a concurrent suspicion of malignancy. Patients referred to an FDG-PET/CT with clinical suspicion of conditions other that PMR/GCA were excluded from the study.

Patient history, clinical signs and symptoms, laboratory results, the suspected diagnosis upon referral to FDG-PET/CT, FDG-PET/CT findings, and the final diagnosis after full diagnostic work-up were registered for each patient based on review of patient records. All solid and hematological malignancies, except for non-melanoma skin cancer, were registered as a malignant outcome. Non-melanoma skin cancers are common, but usually do not metastasize and are not likely to cause B-symptoms. For this reason, non-melanoma skin cancer was not included in our analysis.

The local research ethics committee evaluated the project and did not find ethical approval necessary (J.no. F-23029632). Project approval was obtained by the legal department at Rigshospitalet, Copenhagen.

### Statistics

Descriptive statistics were applied to determine frequencies of different characteristics in the study population. To determine differences between the patients with and without malignancy, student’s T-test and Mann–Whitney U test were used as appropriate on continuous variables, and Fisher’s exact test was used in comparison of binary variables. Statistics were performed using IBM SPSS 28.0.0.0.

Cancer incidences from Denmark across all locations, except for non-melanoma skin cancer, were extracted from the NORDCAN^1^ database. A weighted mean incidence was calculated based on incidences according to the age and sex distribution in the FDG-PET/CT-cohort, in order to determine an estimated standardized incidence ratio (SIR). Confidence intervals were calculated using the Vandenbroucke method.

## Results

In total 314 FDG-PET/CT examinations were identified in the inclusion period. All examinations were screened, and 92 of the patients were referred with other provisional diagnoses than PMR or GCA, and thus excluded from this study.

In 222 of the FDG-PET/CT examinations the referral diagnosis was PMR, GCA or both. Two of the patients had undergone two FDG-PET/CT examinations in the study period, and in these two cases, we chose to include the first exam, as the second one did not provide any additional information. Thus, a total of 220 FDG-PET/CT examinations were included. Most of the examinations were performed as FDG-PET combined with a low-dose computed tomography (FDG-PET/ldCT), but 40 (18.2%) of the examinations were performed with a diagnostic computed tomography (dCT), including the use of an intravenous CT contrast agent. All scans were performed as a conventional whole-body PET/CT scan; from vertex to mid-thigh, including arms, which were positioned along the trunk with hands flat on the bed. Findings suspicious of malignancy were reported in 19 (8.6%) of the exams, and a definite malignant diagnosis was confirmed in 7 (3.2%) cases, of which two were priorly known malignancies. Cohort characteristics and descriptive statistics are summarized in Table 1. The cohort consisted of 146 (66.4%) female patients and 74 (33.6%) male patients. The mean age of all patients was 69.8 years. The patients that were not diagnosed with malignancy had a mean age of 69.6 years, and the group of patients in whom malignancy was confirmed had a mean age of 76.6 years. However, this difference did not reach statistical significance (*p* = 0.07). In the group of patients with confirmed malignancy, 71.4% had a symptom duration of more than 3 months at the time of FDG PET/CT, versus only 39.4% of the patients that were not diagnosed with malignancy, though not statistically significant. There were no significant differences in the mean C-reactive protein (CRP) levels or the mean hemoglobin levels between the patients with and without malignancy (Table 1).

**Table 1:** Patient characteristics at time of FDG-PET/CT.

Characteristic	Total *N* = 220	Patients not diagnosed with malignancy *N* = 213	Patients diagnosed with malignancy *N* = 7	Value of *p*
Age				
Mean (SD)	69.8 (10.1)	69.6 (10.1)	76.6 (7.7)	0.07
Range	45–93	45–93	63–85	
Sex				
Male, n (%)	74 (33.6)	70 (32.9)	4 (57.1)	0.23
Female, n (%)	146 (66.4)	143 (67.1)	3 (42.8)	
Duration of symptoms^1^				
≥ 3 months	89 (40.5)	84 (39.4)	5 (71.4)	0.21
< 3 months	84 (38.2)	83 (39.0)	1 (14.3)	
Unknown	47 (21.4)	46 (20.9)	1 (14.3)	
Biochemistry				
CRP, mean (SD)	36.6 (46.8)	36.3 (46.9)	44.5 (45.0)	0.75
Hemoglobin, mean (SD)	8.04 (0.9)	8.05 (0.9)	7.87 (1.4)	0.60
Suspected diagnosis upon referral to PET/CT				
PMR, n (%)	83 (37.7)	81 (38.0)	2 (28.6)	0.71
GCA, n (%)	31 (14.1)	29 (13.6)	2 (28.6)	0.26
PMR and GCA, n (%)	41 (18.6)	41 (19.2)	0 (0)	0.35
Malignancy^2^, n (%)	60 (27.3)	57 (26.8)	3 (42.9)	0.39
Other^3^, n (%)	5 (2.3)	5 (2.3)	0 (0)	1.00

1 Symptoms: pain and stiffness of shoulder- and/or pelvic girdle, joint pain, headaches, jaw claudication, visual disturbances, weight loss, fatigue, nights sweats and/or fever.

2 Includes suspected PMR and/or GCA + malignancy.

3 Includes suspected PMR and/or GCA + infection/arthritis/polymyositis/other pathology.

PMR, Polymyalgia rheumatica; GCA, Giant cell arteritis.

### Referral diagnoses and symptoms

The clinical suspicion at referral to FDG-PET/CT (Table 1) was solely PMR in 83 (37.7%) patients, solely GCA in 31 (14.1%) patients, and PMR with GCA in 41 (18.6%) patients. Sixty (27.3%) patients were referred with a suspicion of malignancy concomitantly with PMR, GCA or both. In 42.9% of patients with malignancy, there was a suspicion of malignancy upon referral to FDG-PET/CT. Among patients without malignancy, only 26.8% were referred to FDG-PET/CT with a suspicion of malignancy. However, the difference in the pattern of referral diagnoses between the patients with and without malignancy was not statistically significant.

The symptoms leading to the referral to FDG-PET/CT included pain in shoulder and hip girdles as well as in the proximal muscles, swollen joints, weight loss, fatigue, night sweats, headaches, jaw claudication, fever, and/or visual disturbances. There were no statistically significant differences in symptoms, including the frequency of constitutional symptoms, between the patients with and without malignancy, however data from patient records was incomplete in relation to this issue.

### Findings in patients without malignancy

In 120 (54.5%) of the exams there was an enhanced FDG-uptake at PMR predilection sites. In 20 (9.1%) of the scans signs of vasculitis was found. In 19 (8.6%) of the scans there were findings suspicious of malignancy (Table 2).

**Table 2:** FDG-PET/CT findings.

Findings	N (scans)	%
Enhanced FDG-uptake in some PMR predilection sites Fulfills PET-criteria for PMR	120 54	54.5
Vasculitis Aorta and thoracal branches Cranial arteries	20 15 11	9.1
Intraarticular inflammation	39	17.7
Suspected malignancy	19	8.6
Suspected infection	8	3.6

PMR, Polymyalgia rheumatica.

Table 3 summarizes the final diagnosis after completion of the full diagnostic process. In 102 (46.4%) cases the final diagnosis was PMR, while GCA in 31 (14.1%) cases. Rheumatoid arthritis was diagnosed in 15 (6.8%) patients, and 36 (16.4%) patients received other (rheumatic as well as non-rheumatic) diagnoses. In 29 (13.2%) cases, the diagnosis was unresolved.

**Table 3:** Final diagnosis.

	N	%	Comments
Malignancy	7	3.2	*Includes 3 patients with a diagnose of malignancy + PMR, 1 patient with malignancy + RA.*
PMR	102	46.4	*Includes 4 patients with the diagnosis of PMR + RA, 1 patient with PMR + CPPD and 1 patient with PMR + aplastic anemia.*
GCA	31	14.1	*Includes 8 patients with GCA + PMR.*
Rheumatoid arthritis	15	6.8	*Includes 1 patient with RA + gout*
Other	36	16.4	*Includes diagnoses of different non-inflammatory musculoskeletal conditions, eye diseases, psoriatic arthritis, unspecified polyarthritis, fibromyalgia, crystal arthritis, unresolved tumors, endocarditis, vascular claudication, unspecified infection, pleura-pericarditis, Granulomatosis with polyangiitis and polymyositis.*
Unresolved	29	13.2	

PMR, Polymyalgia rheumatica; GCA, Giant cell arteritis; RA, Rheumatoid arthritis; CPPD, Calcium Pyrophosphate Deposition.

### Findings suspicious of malignancy

In total 25 findings suspicious of malignancy were reported in 19 scans (Table 4). In seven cases (3.2%), malignancy was confirmed, of which five (2.3%) were newly diagnosed solid cancers (lung cancer, kidney cancer, breast cancer and two cases of colorectal cancer), and two were related to already known malignancies. In four of the cases, the patients received a rheumatological diagnosis concomitantly with the malignant disease (three patients with PMR and malignancy and one patient with rheumatoid arthritis and malignancy).

**Table 4:** Cancer-suspicious findings on FDG-PET/CT.

Site of suspicious finding	Outcome
Parotid gland (2)	1 Malignancy dismissed
	2 Malignancy dismissed
Thyroid gland (3)	1 Malignancy dismissed
	2 Malignancy dismissed
	3 Malignancy dismissed
Mamma (1)	Breast cancer confirmed
Costa (1)	Not examined further
Thoracal vertebrae (1)	Malignancy dismissed
Lung (3)	1 Lung cancer confirmed
	2 Malignancy dismissed
	3 Malignancy dismissed
Kidney (2)	1 Kidney cancer confirmed
	2 Follow-up still ongoing at time of study end
Retroperitoneal process (1)	Follow-up still ongoing at time of study end
Colon (5)	1 Colorectal cancer confirmed
	2 Colorectal cancer confirmed
	3 Malignancy dismissed
	4 Not examined further
	5 Not examined further
Rectum (2)	1 Malignancy dismissed
	2 Malignancy dismissed
Iliac bone (1)	Malignancy dismissed
Gluteal region (1)	Sarcoma confirmed (priorly known)
Spleen and bone marrow (1)	Myelodysplastic syndrome confirmed (priorly known)
Skin (1)	Basal Cell Carcinoma

In two of the suspicious findings, malignancy could not be confirmed or ruled out with certainty. In both cases, the patients were regularly monitored with imaging, and follow-up was ongoing at time of study-end. Two cases of possible intestinal polyps with enhanced FDG-uptake were not subjected to further follow-up based on the decision of the treating physician, and one finding in a costa was not investigated further due to patient wish. One case of non-melanoma skin cancer was found in a patient with solid cancer and was not included in the data analysis.

An estimated SIR for cancer for the total cohort and stratified by sex was calculated as the ratio between the actual and expected number of malignancies in our cohort. The expected number was based on the sex and age-matched incidence of cancer in the background population in Denmark in 2020^2^. The SIR for the total cohort was 1.58 (95% CI 0.63–2.97), while for men 2.19 (95% CI 0.73–4.42) and for women 1.33 (95% CI 0.35–2.94), and thus, the slightly higher incidence of malignancy in our cohort compared to the expected, was not statistically significant.

## Discussion

In this retrospective study, we found malignancy on FDG-PET/CT in only 7/220 patients referred for FDG-PET/CT as part of the diagnostic work-up for suspected PMR, GCA or both. In 3/7 cases where malignancy was established, this diagnosis occurred concomitantly with PMR, and the total cancer incidence in the cohort did not statistically significantly differ from a sex and age matched background incidence in Denmark. The patients in our cohort with malignancy were numerically older, as compared to patients without malignancies, and most of them had had symptoms for more than 3 months, though the differences were not statistically significant.

Whereas existing studies have focused on malignancy in patients with *diagnosed* PMR or GCA, the current study investigated the frequency of malignancy in patients with *suspected* PMR and/or GCA, and only approximately 60% of the patients in our cohort were ultimately diagnosed with PMR and/or GCA. Thus, our data reflects a real-life diagnostic setting, in which the clinician might consider malignancy as a differential diagnose to PMR/GCA as well as the aspect of PMR/GCA as possible paraneoplastic conditions.

Several studies have examined the relationship between established PMR/GCA and malignancy. Ji et al. and Muller et al. found an increased risk of cancer within the first 6 to 12 months after the diagnosis of PMR and GCA in large-scale register-based studies (20, 21). Similarly, Dar et al. and Bellan et al. both found an increased risk of cancer in patients with GCA and PMR, respectively, and both studies found that male sex and older age were independent predictors for malignancy (16, 22). Conversely, other studies, such as those from Pfeifer et al. and Hill et al. have not been able to confirm a higher risk of malignancy in patients diagnosed with PMR/GCA (23, 24).

Two recent prospective studies have addressed the issue of systematic examination for malignancy in patients with PMR/GCA. Ramon et al. examined patients who met the 2012 ACR/EULAR classification criteria for PMR with a diagnostic computed tomography of the thorax, abdomen and pelvis (dCT-TAP) and found a frequency of malignancy of 7.6% and an SIR of 4.63 compared to the background population. They did not find differences in age, disease duration, symptoms, or inflammatory marker levels in patients with and without malignancy (17). Emamifar et al. examined patients with PMR/GCA with FDG-PET/CT and found a frequency of solid cancers of 5.2%. They found that patients with solid cancers were older than the patients without cancer (25). These frequencies are higher than in the present study. However, the patients in these studies were already diagnosed with PMR/GCA, and the population is thus different from ours which comprises patients suspected of PMR/GCA. Another potential reason for the higher frequency found by Ramon et al. may be the difference in imaging modality. However, similar results were found in a recent study of patients with large vessel vasculitis, including GCA, on FDG-PET/CT. Tumors were found in 7.2% of the patients, though it is not reported whether these were all malignant or also included benign tumors (26).

As healthy people without symptoms of, e.g., malignancy rarely undergo FDG-PET/CT examinations, the frequency of malignancy as incidental findings on FDG-PET/CT in a normal population is not known. Wan et al. found cancer as an incidental finding on PET/CT in 6/259 (2.31%) otherwise healthy patients with moderate-to-severe psoriasis (27). These results are quite comparable to ours, especially when taking into consideration that the mean age in their cohort was lower (45.3 years).

A strength of this study is that it is based on individual patient chart reviews, as opposed to registry-based studies, in which wrongful categorizations might occur. Limitations of this study include the relatively small sample size, the retrospective design which entails some incomplete data, as well as the lack of an actual control group. A formal control group of healthy patients regarding FDG-PET/CT is ethically very difficult to obtain. Furthermore, there is a risk of selection bias, as not all patients evaluated for PMR/GCA in the study period would have undergone an FDG-PET/CT, and that patients with atypical presentations, would probably be more likely to be referred to an FDG-PET/CT.

In conclusion, this retrospective study found a total frequency of malignancy of 3.2% in PMR/GCA suspected patients referred to an FDG-PET/CT, and almost half of these patients received a concomitant diagnosis of PMR/GCA. Thus, malignancy as the solitary cause of the patients’ symptoms was infrequent in the current study, and the observed number of detected malignancies in the cohort did not exceed the expected number in the background population with statistical significance. Our results, though based on a small cohort, do not suggest that all patients with suspected PMR/GCA should systematically be examined with FDG-PET/CT for excluding malignancy.

## Data availability statement

The datasets presented in this article will be made available by the authors upon reasonable request within the scope of the research project’s legal approval. Requests to access the datasets should be directed to tanja.fromberg.gorlen@regionh.dk.

## Ethics statement

Ethical approval was not required for the study involving humans in accordance with the local legislation and institutional requirements. Written informed consent to participate in this study was not required from the participants or the participants’ legal guardians/next of kin in accordance with the national legislation and the institutional requirements.

## Author contributions

TG: Formal analysis, Investigation, Writing – original draft, Writing – review & editing, Conceptualization. JMB: Writing – review & editing, Conceptualization. MØ: Supervision, Writing – review & editing, Resources. BF: Writing – review & editing. UD: Conceptualization, Methodology, Writing – review & editing. LT: Conceptualization, Methodology, Supervision, Writing – review & editing.

## Footnotes

^1^ https://nordcan.iarc.fr/en

^2^ https://nordcan.iarc.fr/en
